# The underexplored links between cancer and the internal body climate: Implications for cancer prevention and treatment

**DOI:** 10.3389/fonc.2022.1040034

**Published:** 2022-12-22

**Authors:** Doru Paul, Aurora M. Nedelcu

**Affiliations:** ^1^ Weill Cornell Medicine, New York, NY, United States; ^2^ Biology Department, University of New Brunswick, Fredericton, NB, Canada

**Keywords:** cancer, homeostasis, prevention, treatment, systemic disease, internal climate

## Abstract

In order to effectively manage and cure cancer we should move beyond the general view of cancer as a random process of genetic alterations leading to uncontrolled cell proliferation or simply a predictable evolutionary process involving selection for traits that increase cell fitness. In our view, cancer is a systemic disease that involves multiple interactions not only among cells within tumors or between tumors and surrounding tissues but also with the entire organism and its internal “milieu”. We define the internal body climate as an *emergent property* resulting from spatial and temporal interactions among internal components themselves and with the external environment. The body climate itself can either prevent, promote or support cancer initiation and progression (top-down effect; i.e., body climate-induced effects on cancer), as well as be perturbed by cancer (bottom-up effect; i.e., cancer-induced body climate changes) to further favor cancer progression and spread. This positive feedback loop can move the system towards a “cancerized” organism and ultimately results in its demise. In our view, cancer not only affects the entire system; it is *a reflection of an imbalance of the entire system*. This model provides an integrated framework to study all aspects of cancer as a systemic disease, and also highlights unexplored links that can be altered to both *prevent* body climate changes that favor cancer initiation, progression and dissemination as well as *manipulate* or *restore* the body internal climate to hinder the success of cancer inception, progression and metastasis or improve therapy outcomes. To do so, we need to (i) identify cancer-relevant factors that affect specific climate components, (ii) develop ‘*body climate biomarkers*’, (iii) define ‘*body climate scores*’, and (iv) develop strategies to prevent climate changes, stop or slow the changes, or even revert the changes (climate restoration).

## 1 Introduction

### 1.1 Premise

Despite increasing research efforts, our understanding of cancer as a disease and our ability to cure or manage it are still progressing slower than anticipated. The American Cancer Society Facts and Figures annual report is sobering. Almost 40% of people in United States can expect a cancer diagnosis at some point in their lives, and despite significant investment in research for cancer therapies, only 65% of them will survive for longer than five years after diagnosis (1). More than 50 years after Nixon declared the famous war on cancer, still more than 600,000 people die yearly of cancer in United States (1).

A plethora of new ideas, views and frameworks have been proposed (see (2) for a synthesis) to address the emergence and progression of cancer. Many of the current views are using field-specific frameworks that are usually assumed to either apply to all aspects of cancer or are discussed in isolation. For instance, mutation-based views are focused on identifying specific driver mutations responsible for tumor initiation as well as cancer progression (immune evasion, drug resistance) with the goal of designing targeted and personalized therapies. On the other hand, most evolutionary views consider mutations as the substrate on which selection can act and see cancer as an evolutionary process that can be predicted and even altered. However, although evolutionary theory has been successfully used to understand tumor progression and the emergence of drug resistance, its applicability to other aspects of the disease (e.g., metastasis, cachexia) is not as clear. Furthermore, within the evolutionary framework, the main focus is still mainly on mutational changes (genetic or epigenetic), their dynamics and the intrinsic fitness benefits (e.g., increased cell proliferation, death and immune evasion) they might confer to different cancer clones. Ecological principles are also starting to be applied to understand tumor progression [i.e., the concept of tissue and tumor microenvironments) and metastasis [i.e., the “seed and soil” hypothesis (3)]. However, the focus is mainly on the local environment (i.e., primary or secondary tissue) (4–6). Nevertheless, recent studies are emphasizing the fundamental role that communication between the primary tumor and other organs (i.e., bones) appears to play both in the metastatic process – through the formation of the metastatic niche, as well as in cachexia (7–9). Additionally, sociological/behavioral concepts – highlighting interactions between cells (cooperation, cheating) have been used to understand cancer’s evolutionary origin (as a breakdown of multicellular cooperation) as well as tumor progression and metastasis (i.e., interclonal cooperation). At the other end, views that are built on the framework of reversal to embryonic states (de-differentiation) or to early stages in the evolution of multicellularity or the eukaryotic cell itself (atavism) emphasize the activation of existent genetic programs without the need to specifically invoke evolutionary or ecological principles (see (10) for discussion and references).

### 1.2 The problem

Cancer is a complex disease that requires a multifaceted and integrated approach to understand and manage all its aspects. Building on Claude Bernard’s concept of “milieu interne” (11), Walter Cannon’s concept of homeostasis (12) and James Hardy’s concept of set points (13), we introduce here the concept of body “climate”. In our view, all organisms are characterized by an internal body climate, which is a pervasive element that (i) is the resultant of the activities of all the different elements of the body (the cells, the tissues and the whole organism) and their interactions (bottom-up effects) as well as the level where are all these changes are integrated, and (ii) affects the functioning of all these embedded elements (top-down effects) to maintain the homeostasis of the system. These activities are fully analogous to the functioning of natural systems involving interactions among different components of the biosphere and with the climate, in terms of both how the biosphere can change the climate and how the climate can affect the biosphere.

In this framework, cancer is not only a disease of the cell (the *seed*; used here to refer to both the initiating and dispersal cells). Likewise, cancer is not only affected by, and it does not only affect, the tissue (the *soil*; both primary and secondary). The entire organism can indirectly influence cancer initiation (*seed germination*) and progression by affecting tissues (the *soils*) *via* systemic factors. And tumors (*opportunistic weeds*) can also directly perturb not only the tissues (*soils*) or organs (*ecosystems*), but also the entire system (*biosphere*) and its climate. The body climate, in turn, can affect other tissues and thus the potential for successful metastasis (secondary *seed establishment*) and result in the demise of the system. Ultimately, cancer is a systemic/climate disease both in terms of its causation and its consequences.

### 1.3 Our proposal and approach

We propose that cancer should be considered in the context of the entire system – both from the point of view of how systemic climate changes associated with physiological and metabolic perturbations, diet or age (*body climate-induced changes*) can prevent, induce or affect various aspects of cancer initiation, progression and dispersal (top-down effects), as well as how cancer can affect not only the residing or surrounding tissue, but also the functioning and the climate of the entire organism (*cancer-induced body climate changes*; bottom-up effects). These interactions have important consequences (through positive feedback-loops) for the metastatic process and the ultimate breakdown of the system (i.e., cachexia and death). In our view, cancer not only affects the entire system; it is a reflection of an imbalance of the entire system. Specifically, cancer is a reflection of changes in the internal climate of the system; and cancer affects the entire body climate.

This model not only provides an integrated framework to study all aspects of cancer as a systemic disease, but can also highlight unexplored links that can be altered to both prevent climate changes that favor cancer initiation, progression and dissemination as well as reconstitute or manipulate the internal climate to hinder cancer initiation, progression and metastasis or improve therapy outcomes. The goal of this approach is to shift the focus from mutations and changes in cell phenotype/behaviour or tissue microenvironments to entire body climate. To do so, we need to (i) identify relevant ‘*body climate factors and components*’ relevant to cancer initiation and progression, (ii) develop ‘*body climate biomarkers*’, (iii) define ‘*body climate scores*’, and (iv) develop strategies to *prevent ‘body climate change’*, stop or slow the changes, or even revert the changes (‘*climate reconstitution’*). Here, we provide an overview of the framework we need to develop in order to fully understand and manage cancer as a systemic disease, as well as some (non-exhaustive) examples of the complex issues and aspects that need to be considered to achieve those goals.

## 2 The body’s internal climate

The importance of the body’s internal “milieu” was first noticed by Walter C. Canon who wrote in “The wisdom of the body” (12): “stable states for all parts of the organism are achieved by keeping uniform the natural surroundings of these parts, their natural environment or *fluid matrix*. That is the common *intermedium*, which, as a means of exchange of materials, as a ready carrier of supplies and waste, and as an equalizer of temperature, provides the fundamental conditions that facilitate *stabilization* in the different parts. The central problem in understanding the remarkable stability of our bodies, therefore, is that of knowing how the uniformity of the fluid matrix is *preserved*”.

**Figure 1:**
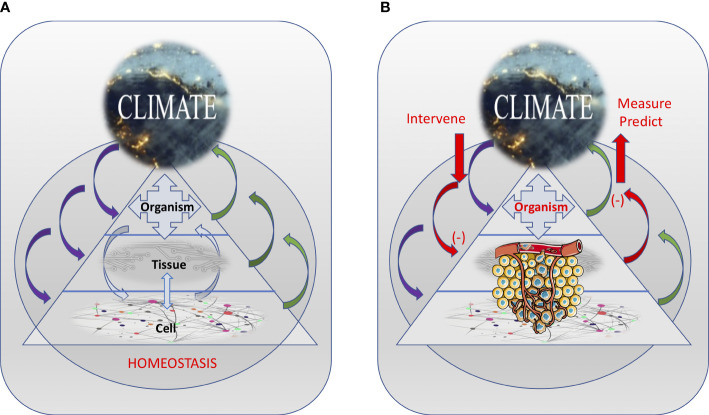
The inter-dependence among the various interacting body levels and the internal climate, resulting in maintaining the homeostasis of the system **(A)** or promoting cancer development **(B)**. Internal climate changes induced by cancer can be measured and used to predict changes in the system. Also, external interventions can be used to alter the internal climate in ways that can prevent or slowdown cancer development. Tumor illustration from Servier Medical Art - https://smart.servier.com.

This “milieu interne”, an expression coined by Charles Robin and used subsequently by Claude Bernard, confers the body a certain independence in relationship with the external environment: “This kind of independence which the organism possesses in the external environment derives from the fact that within the living being the tissues are in fact removed from direct external influences and are protected by a veritable internal environment which is constituted particularly by the fluids which circulate in the body [ … ]. In humans and in warm-blooded animals, the independence of the external environment and the internal environment is such that one could consider these beings as living in their own organic environment” (11). In our view, however, the internal body climate is more than just the “fluids which circulate in the body”; it is a pervasive systemic element that is influenced by, as well as influences, all levels – from cells and tissues to organs and the entire organism (**Figure 1A**).

These integrated, complex and dynamic interactions within the body can be likened to the interactions that determine (and can be determined by) the climate in natural systems. Specifically, similar to how interactions among various climate factors (such as the sun, the earth, the oceans and the seas, the atmosphere and all life forms) reflect in several climate components (like radiation, air pressure, temperature, humidity, wind speed), interactions among body factors (e.g., cells, tissues, organs, body structure, and microbiota) can affect (and be affected by) the body’s internal climate components (i.e., internal fluid composition, pressure, temperature, pH, electrical charge and biorhythm).

**Figure 2:**
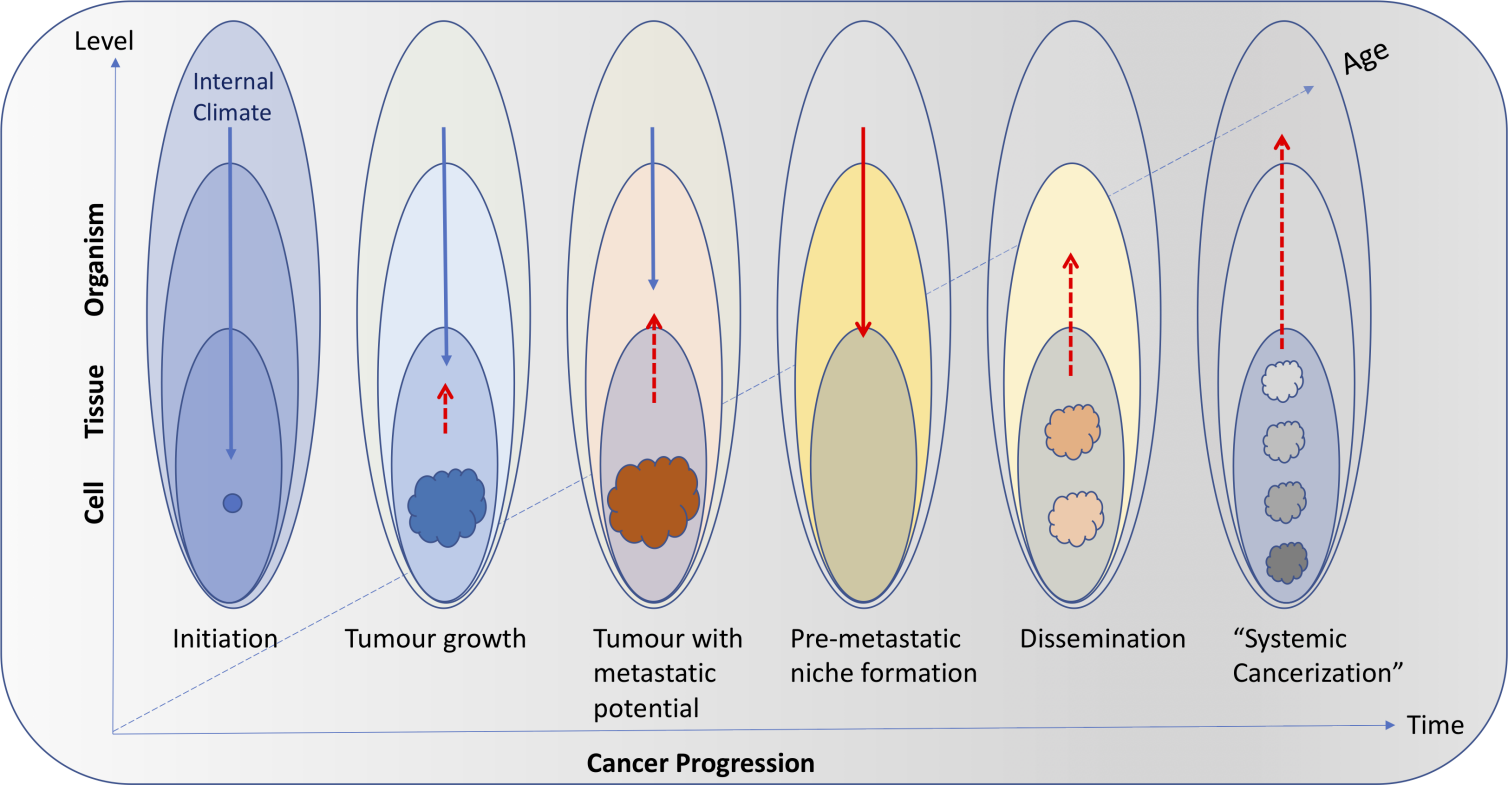
Interactions among body levels (cell, tissue, organism) and the internal climate can change with time as cancer progresses as well as a function of age and changes in the life-style of the individual. In young and/or healthy individuals, the internal climate can have a strong suppressing role on cancer initiation (blue arrows). As age and/or cancer progresses, tumors (coloured masses) can increasingly affect the climate (red dashed arrows) and the suppressing role of the internal climate diminishes. In advanced disease, the internal climate can even have a facilitating role (solid red arrow) that will result in promoting tumor growth and cancer dissemination through pre-metastatic niche formation. The new tumors (pink masses) will continue to affect the climate and the positive feedback loop will ultimately drive systemic cancerization (gray and black masses).

Also, as in natural systems, the body climate has a temporal and spatial dimension. For instance, the body climate can changes with age (i.e., the body climate of a teenager is different from the body climate of a nonagenarian), physiologic state (e.g., pregnant woman vs. non-pregnant woman), and in response to the external climate (e.g., pollutants, tobacco, sun exposure). Different cancers developing in different tissues and organs may be related to very different changes in their local climate characteristics. But the organismic level – through systemic factors (e.g., damaging agents, metabolites, hormones), can directly (by affecting cells) or indirectly (by affecting tissue health) suppress or promote cancer initiation and progression. Similarly, tumors can directly or indirectly (e.g., through factors released in the circulation) affect resident and distant tissues (e.g., during metastasis) as well as the entire organismic climate (e.g., during cachexia) (**Figure 1B**). Importantly, these various climate factors and the way they interact with each other also change over time (in a temporal context) both as cancer progresses as well as a function of the age and changes in the life-style of the organism (**Figure 2**).

Overall, the utility of the internal body climate concept (in contrast to other more abstract concepts) is that it encompasses a series of systemic components that can be measured and whose fluctuations reflect systemic changes. Furthermore, the effects of changes in the body climate components can be predicted. However, in contrast to Earth’s climate, the body climate is not a passive, un-regulated component in the organism’s physiology. Both body climate factors and components are under homeostatic control.

## 3 Body climate: factors and components relevant to cancer

**Figure 3:**
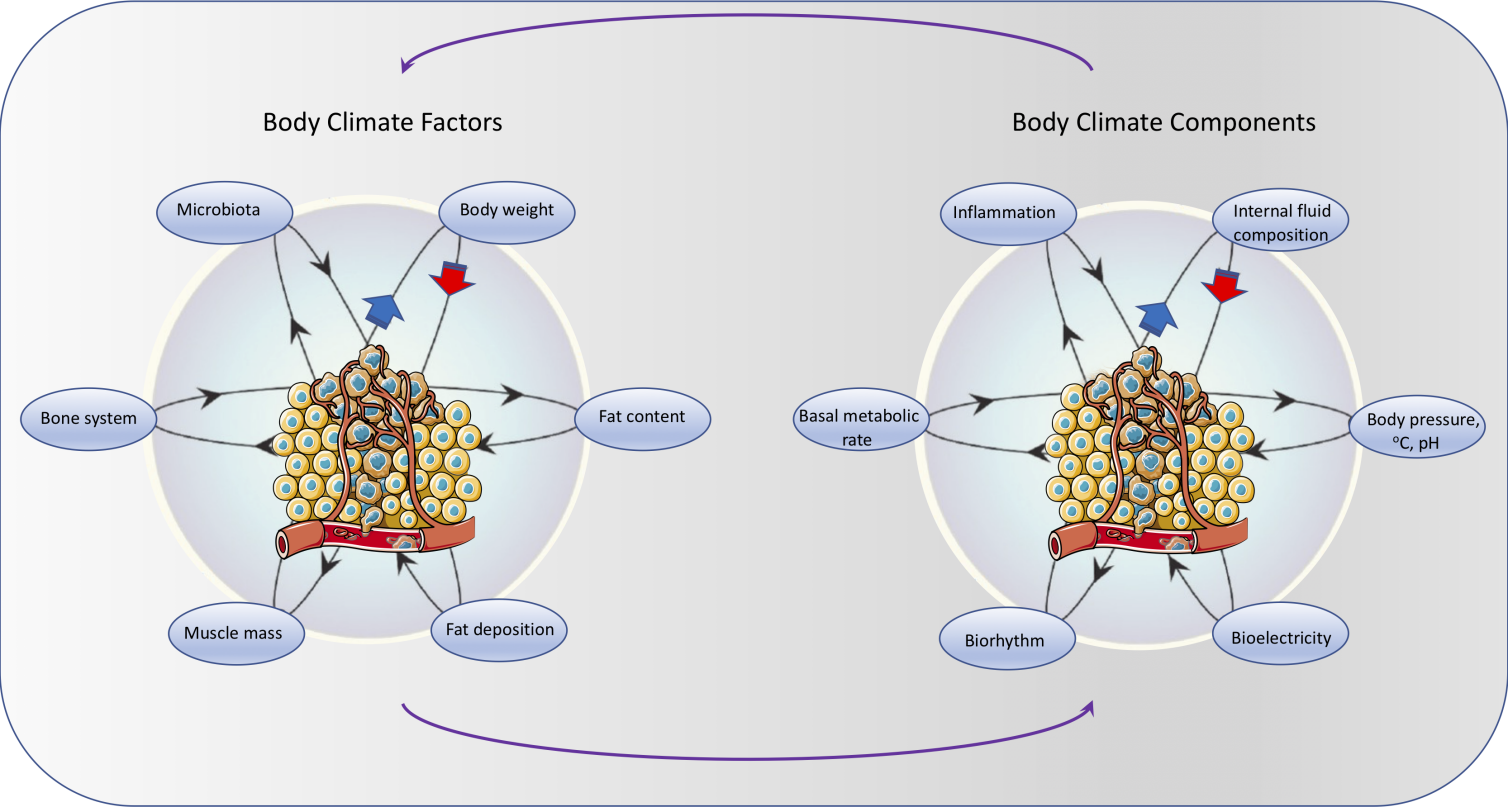
Similar to natural climate systems, several interdependent intrinsic climate factors – such as body structure (fat, muscle, bone, microbiota) affect a number of climate components (internal fluid composition, body pressure, temperature, pH, electric potential, biorhythm, basal metabolism rate, inflammation). In addition, cancer itself can directly or indirectly affect body climate factors and components. Tumor illustration from Servier Medical Art - https://smart.servier.com.

Several interdependent factors contribute to, and affect, the body’s internal climate. They include both intrinsic factors – such as body structure (fat, muscle, bone, microbiota) and extrinsic factors – such as diet and external environment (sun, tobacco, exposure to pathogens or mutagens). These factors can influence several internal climate components, including the internal fluid composition, body pressure, temperature, pH, electric potential, biorhythm, basal metabolism rate, and inflammation. In addition, cancer itself can directly or indirectly affect body’s climate factors (e.g., body weight, muscle mass, fat content, bone system, microbiota) and internal climate components (e.g., pH, electric potential, inflammation) and can be affected by the internal climate (**Figure 3**).

Below we provide an overview of the main internal climate factors and components that affect and define the organismic climate, both in normal conditions as well as in relation to various aspects of cancer development. We focus more on the climate components because, by definition, they describe the climate and can be affected by a multitude of interactions (and changes) among climate factors. Also, due to their highly dynamic nature (relative to climate factors), changes in their levels can be assessed/measured and be used as indicators of climate status, which in turn can have a prognostic or diagnostic value.

### 3.1 Internal factors that can influence the body climate

#### 3.1.1 Body weight and fat deposition/distribution can contribute to an inflammatory climate

Body weight emerged as an important factor in the development of cancer. In United States, among people aged 30 and older, it has been estimated that between 2011-2015, about 37,670 new cancer cases in men (4.7%) and 74,690 new cancer cases in women (9.6%) were due to excess body weight (overweight, obesity, or severe obesity) (14). There is consistent evidence that twelve cancer types (15) – ovarian (16), breast (17, 18), endometrial (19), thyroid (20), gallbladder (21), colorectal (22), pancreatic (23), gastric (24), esophageal (25), liver (26), kidney (27) and multiple myeloma (28), are related to excessive body fat.

Interestingly, the risk seems to be determined not only by excessive fat accumulation *per se* but also by the location of fat deposition (with increased risk for the intra-abdominal fat deposition; i.e., central obesity) (29). Epidemiologic evidence shows that the shape of the body (high waist-to-hip ratio, WHR; e.g., apple-shape vs pear-shape) is also important (30). For instance, people who carry most of the fatty tissue in the abdominal region are 70% more prone to develop pancreatic cancer compared to those who bear it around the hips (29); and an apple-shaped body is associated with a higher risk of colorectal cancer in men but not in women (31). Also, a large Chinese study (32) that enrolled 1316 women demonstrated that women with more fat around their thighs, hips, and buttocks had a higher risk of hormone-receptor-positive breast cancer compared to women with less fat in these locations.

These findings may be explained by the different metabolic profile of subcutaneous fat compared to visceral fat (33). Visceral adipose tissue was shown to be more prone to generate inflammation – an important climate component (discussed later).

#### 3.1.2 Muscle mass can act as an endocrine organ and have beneficial systemic effects on the internal climate

Skeletal muscle can play an active role in modifying body`s internal climate. Overweight patients although at an increased risk for cancer often have a paradoxically lower risk of overall mortality after a cancer diagnosis, a phenomenon called the “obesity paradox” (34). The apparent paradox may be explained by the fact that, in addition to an excess of fat, overweight patients may also have the necessary protective muscle reserves. Recently, a high level of muscle mass was associated with improved overall survival in prostate cancer patients regardless of treatment stage (35). Over the last decade, it has been demonstrated that skeletal muscle works as an endocrine organ, which can produce and secrete hundreds of myokines that may have a *systemic effect* on lipid and glucose metabolism, browning of white fat, and tumor growth (36, 37).

#### 3.1.3 Bone can affect and be affected by the internal climate, with consequences for cancer development

As demonstrated by recent studies, bone is not a static organ and, similar to the muscle, also behaves like an endocrine gland secreting several hormones that regulate energy metabolism and reproduction (38). High serum levels of osteocalcin, a hormone secreted by the bone (39, 40) have been linked to the development of prostate cancer. Bone also plays a key role in cancer progression. Several *in vitro* and *in vivo* experiments have shown that metastatic cancer cells, upon arrival to bone, interact with osteoblasts on the endosteal surface, which in turn maintain cancer cells in a dormant state by inhibiting their proliferation. In contrast, osteoclasts play an opposite role and may reactivate dormant cancer cells (36). Furthermore, osteopontin released from primary “instigating” tumours into circulation has been shown to activate and mobilize bone marrow cells that can travel to distant location and induce the growth of indolent tumours (37).

#### 3.1.4 Microbiota – a microscopic climate factor with major sensitivity to, and influence on, several climate components

Gut microbiota has been shown to have an important contribution to the overall health status of individuals, through systemic effects, especially with respect to obesity and related metabolic diseases (41). Importantly, microbiota is strongly affected by diet (i.e., an extrinsic climate factor), which modulates the activity of host and gut microbiota synchronously to influence their interaction. For example, a high-fat diet enhanced gene expressions of both the host and microbiota (42, 43). The gut microbiota could induce or modulate the signal transmission directly or indirectly to affect energy homeostasis (44).

Microbiota present in the gut is under a different type of homeostatic control called “commensal homeostasis” (45). Recently, it has been proposed that the host regulates microbiome homeostasis, which allows for the possibility that imbalances in gut microbiome can be monitored through measuring host parameters such as the oxygen and nitrate concentration along the longitudinal axis of the intestine, and the quantity and composition of bile acid metabolites (46). However, microbiota can also directly affect cancer progression and therapy (47); for instance, during androgen deprivation therapy, gut microbiota can produce androgens that are absorbed into circulation and promote prostate tumor growth (48).

### 3.2 Body climate components that can affect and are affected by cancer

The internal body climate can be defined by a multitude of components that reflect interactions among various elements of the body (intrinsic climate factors: cells, tissues, organs, microbiota, and cancer) as well as between the body and the external environment (including diet – discussed later). Below we provide an overview of some of the internal climate components that reflect interactions relevant to cancer. In the sections on cancer prevention and cancer treatment we focus on the ways these specific internal climate components can be manipulated to prevent or slow the progression of cancer.

#### 3.2.1 Internal fluid composition – a complex and dynamic internal climate component

The composition of internal fluids (blood, lymph, interstitial fluid) is a premier example of an internal climate component as it is the result of various processes that reflect the functions of multiple internal climate factors and their interactions with the external environment. But this complex climate component can also be affected by, and affect, cancer progression – from promoting to inhibiting it, directly or indirectly. Some blood parameters are used as general health indicators (body climate markers) and others can be used as specific prognostic or indicators of cancer progression. The interstitial fluid composition, which reflects both the cellular and tissular activity and the blood and lymph composition, has also been implicated in cancer progression and treatment (49).

##### 3.2.1.1 Cellular blood components

Many cellular blood components are employed as indicators of health status, especially with respect to infections (e.g., eosinophils for parasitic infections), inflammation (neutrophils), and cancer progression (circulating tumor cells – CTCs; circulating tumor DNA – ctDNA). Cancer can also induce changes in the levels of various blood cells (i.e., cancer-altered body climate components), which are often used as prognosis indicators. For instance, increased circulating neutrophils is a known adverse prognostic factor in several cancer types (50, 51). Anemia is another independent prognostic factor for survival in patients with cancer (45); and in colorectal cancer the severity of anemia is also associated with disease stage (52). A decreased cytotoxic functionality of NK cells correlates with increased numbers of circulating tumor cells (CTCs) in patients with metastatic breast, colorectal and, prostate cancer (53). Also, platelets can be used by cancer cells to protect them from Natural Killers (NK) cells attack, and may help cancer cells in the metastatic process (54, 55).

##### 3.2.1.2 Macromolecules

Levels of lipids, carbohydrates and proteins in internal fluids are associated, in part, with dietary inputs. Compared to normal cells, tumor cells have a higher demand of precursors for proliferation, and reprogram multiple intracellular metabolic pathways to satisfy these needs (56, 57). Thus, various precursors can promote (but also prevent) cancer development and progression (discussed later in the section on Diet); that is, they act as cancer-promoting or cancer-preventing climate components. For instance, an increase in the serum level of branched chain amino acids (leucine, isoleucine, and valine) may precede with several years the clinical development of pancreatic cancer (58, 59). Besides pancreatic cancer, a disruption of systemic metabolism of branched-chain amino acids has been described in several cancers (60). However, changes in the levels of various macromolecules can also reflect changes in various body activities, including cancer progression (i.e., act as cancer-altered climate components). For instance, cachectic cancer patients have both adipose tissue and skeletal muscle wasting (61). Interestingly, the overall plasma fatty acid levels seem to be maintained and only oleic acid is significantly higher in patients with cachexia (62).

##### 3.2.1.3 Hormones and other active molecules

The levels of circulating hormones are also good indicators of health and internal climate status. Changes in the levels of many hormones have been demonstrated both in animal models and in large epidemiological studies to play a key role in the development of many types of cancer, including breast, prostate, uterine, ovarian, testicular, thyroid and bone cancers (63–72) (**Table 1** and **Supplementary Material**).

**Table 1:** Several hormones and active molecules associated with cancer risk.

Hormone	Change	Effect	Reference
**Thyroid**	Low	Reduced cancer aggressivity and decreased risk of prostate cancer Increased risk of colorectal and hepatocellular carcinoma	(63)
	High	Increased breast cancer risk	(64)
**Insulin**	High	Increased breast, endometrial, ovarian and pancreatic cancers risk; increases cancer mortality, in general	(65)
**Estrogen**	High	Increased risk of both estrogen positive and negative breast cancer	(66) (67)
**Testosterone**	Low	Increased prostate cancer aggressivity	(68)
**Melatonin**	High	Prevents metastases	(69)
	Disregulated	Increases breast cancer risk	(70)
**Epinephrine/norepinephrine**	High	Increased in head and neck cancer	(71)
**Glucocorticoids**	High	Increase breast cancer metastases	(72)

Tumours themselves can also release a series of hormones that can support cancer progression. Production of specific hormones by tumors of particular types is not random (73). For example, squamous cell carcinomas typically produce parathyroid hormone-related proteins, and small cell carcinomas (SCC) of the lung typically produce calcitonin, adrenocorticotropin (ACTH), or gastrin releasing peptide (GRP). In some case for example, bombesin (BBS)-like neuropeptides secreted by SCC can act as autocrine growth factors (74). Furthermore, osteopontin released by primary tumours can instigate the growth of distant indolent tumours (37).

##### 3.2.1.4 Metabolites

Changes in the levels of many metabolites have also been associated with disease promotion (cancer-promoting climate change) or reflect disease progression (cancer-induced climate change). For instance, a nested case-control study of 2,248 women from the European Prospective Investigation cohort found that concentrations of arginine, asparagine and phosphatidylcholines were inversely associated with breast cancer risk, while the acylcarnitine C2 was positively associated with disease risk (70). More recently, variations in levels of glycine, serine, sphyngomyelin and free carnitine were linked to endometrial cancer development (75). Systemic circulating metabolites have also been found in other types of cancer [e.g., lung – (76); kidney – (77), ovarian-(78); colorectal – (79); pancreas – (80); prostate –(81)] pointing to potentially novel biomarkers and treatments. Similarly, methylmalonic acid (MMA), a by-product of propionate metabolism, was found to be upregulated in the serum of older people and functions as a mediator of tumor progression (82). This suggests that MMA targeting may be a novel therapeutic approach for advanced carcinomas (82).

##### 3.2.1.5 Exosomes

Among other activities, exosomes are known to be involved in cancer metastasis and help cancer cells avoid immune attack (83). Specifically, there are several reports describing the involvement of tumor secreted exosomes in metastasis, and many exosome-associated micro RNAs have been described (i.e., miR-105, miR-181c) (84, 85). Thus, exosome-related components either as part of the exosome cargo or on the exosomes surface can also be used as climate markers (86, 87).

##### 3.2.1.6 Circulating PD-L1

PD-L1 is a protein found on the surface of some normal immune cells and in higher-than-normal amounts on some types of cancer cells. When PD-L1 binds to PD-1 (a protein found on T cells), it acts as a sort of “brake”, impeding the ability of T cells to kill PD-L1-expressing cells, including cancer cells. Circulating PD-L1 has been detected in lung cancer, gastric cancer, renal cell carcinoma, melanoma, hepatocellular carcinoma, pancreatic cancer, breast cancer, and soft tissue sarcoma (88, 89). Its significance for cancer progression is currently under investigation. Recently, removal of circulating soluble and PD-L1-positive extracellular vesicles from periphery through plasmapheresis (i.e., a manipulation of the internal climate) has been developed as a therapeutic intervention in melanoma, as an adjunctive of immunotherapy (90).

##### 3.2.1.7 Oxygen

Oxygen is an important climate component whose levels can vary within the body. Local hypoxia is associated with tumor progression, but it is unclear whether blood oxygen levels play any role in patients with cancer. A recent study suggested that in patients with advanced cancer and low oxygen saturation, oxygen use was not significantly associated with survival (91). Interestingly, the body maintains specific oxygen levels in different parts of the gut (i.e., high in the small intestine but low in the colon), which can directly influence gut microbiome composition (aerobic vs fermentative) (46). And changes in the ability to maintain gut microbiome homeostasis has been linked to many diseases, including cancer (discussed later).

##### 3.2.1.8 Microelements

Microelements, although found in extremely low levels, are an important internal climate component that can play complex roles in cancer (i.e., act as cancer-promoting or cancer-preventing climate components); as well as can be affected by cancer. Several examples of such components and their relation to cancer-inducing climates are included in **Table 2** (see Supplementary Material for additional details).

**Table 2:** Several microelements associated with cancer risk.

Microelement	Change	Effect	Reference
**Zinc**	Low	Increased cancer incidence	(92)
	High	Increased metastasis in thyroid cancer	(93)
**Selenium**	High	Lower risk of breast cancer	(94)
**Phosphorus**	High	Lethal and high-grade prostate cancer	(95)
**Calcium**	High	Increased risk of prostate cancer	(96–103)
**Iron**	High	May increase or decrease risk of colorectal cancer according to the nutritional source	(104)
**Iodine**	High	Lower risk of breast cancer	(105)
**Magnesium**	Low	Increases risk of colorectal and pancreatic cancer	(106–108)
**Salt**	High	Increases risk of gastric cancer	(109)
**Manganese**	High	Decreases risk of prostate cancer	(110)
**Iron**	High	May increase or decrease risk of colorectal cancer according to the nutritional source	(104)
**Copper**	Low	Associated with decreased risk of breast cancer metastases	(111)
	High	May decrease zinc in several cancer types	(112)

Cancer itself can also affect the levels of some minerals. For instance, potassium released from dying tumor cells has been found to suppress the activity of T cells of the immune system. Enhancing the removal of potassium from T cells restores their ability to attack cancer (113).

#### 3.2.2 Body pressure can affect cancer progression

The interstitial fluid pressure is determined by the hydrostatic and the colloid osmotic pressures in capillaries and in the interstitium, together with the hydraulic conductivity and the plasma protein reflection coefficient (114). It has been proven for two decades now that interstitial fluid pressure in the tumor tissue is related to survival in some cancer types (115). It has been also shown in xenograft models that lowering the tumor interstitial fluid pressure reduces tumor cell proliferation (116). Also, the increase in the interstitial fluid pressure limits access of drugs into tumor cells distant from the exchange blood vessels and is one of the mechanisms of drugs resistance (117).

Recent studies have shown that both hypertension (118) and cardiac insufficiency (119) may be significant risk factors for different cancer types, and an integrated approach to both conditions has been proposed (120). Strong positive associations were observed between hypertension and breast, kidney, colorectal cancer (121–123). Also, hypertension was found to increase the risk of esophageal adenocarcinoma and squamous cell carcinoma, liver and endometrial cancer, but the majority of studies did not perform comprehensive multivariable adjustments (118). In general, the mechanism behind these associations is unclear. For renal cancer, it has been speculated that chronic renal hypoxia, lipid peroxidation and deregulation of renin-angiotensin system may be involved (118, 124).

Cancer and cardiac insufficiency share several risk factors: diabetes mellitus, smoking, obesity, hypertension; and the common underlying pathophysiological mechanism underlying both conditions may be inflammation (125, 126). An interesting suggestion has been that the “climates” promoting cancer and cardiac insufficiency are similar (127) and, besides inflammation, similar genetic alterations may be related to both some hematological malignancies and cardiac failure. The same authors also suggested that cardiac failure is an oncogenic condition that can directly influence cancer development. Supporting this idea, it was found that the postischemic failing heart stimulates colon tumor growth in a mouse model, through different secreted factors including SerpinA3 (128).

#### 3.2.3 Body temperature can affect and be affected by cancer

The body temperature reflects body metabolic activity and is maintained relatively constant by feedback loops coordinated by two hypothalamic nuclei, the preoptic area and the dorsomedial hypothalamus (129). Thus, body temperature is a good indicator of health, especially in contexts associated with microbial infections and inflammation. But body temperature is also an important climate component that can both be affected by cancer or promote/prevent cancer. For instance, cancer can induce an increase in body temperature (cancer-induced climate change). On the other hand, changes in body temperature can also affect cancer (cancer-promoting or cancer-preventing internal climate changes).

A key preclinical study showed that tumor growth rate and metastatic burden of mice housed at thermoneutral temperature (approximately 30 to 31°C) were significantly reduced compared to those housed at standard temperature (approximately 22 to 23°C) (130). These differences were not seen in immunodeficient mice suggesting that sub-thermoneutral temperatures may affect antitumor immune responses. In the immunocompetent mice, the numbers of antigen-specific CD8^+^ T lymphocytes and CD8^+^ T cells with an activated phenotype in the tumor microenvironment significantly increased at thermoneutrality. Interestingly, the body temperature of the USA population has been declining over the last century (131) and this decrease might have contributed to the concomitant increase in cancer incidence during the same time period. Furthermore, a negative correlation between the average annual environmental temperature and cancer incidence has been reported for the female (and less for the male) population in the USA (132).

#### 3.2.4 Local pH affects cancer progression

Internal fluids are characterized by a relatively constant pH (around 7.4), and changes in pH are generally buffered, especially in the blood. However, local decreases in pH are known, especially in advanced tumors; but these changes are not reflected in the blood pH of cancer patients, which, in general, is not influenced by tumoral pH (133). The low pH of tumor microenvironment is mainly the consequence of cancer metabolic reprogramming resulting in the release of lactic acid as by-product of anaerobic glycolysis (134). The acidic microenvironment promotes cancer growth and metastasis (135, 136). The pH of the local tissue microenvironment can also influence cancer progression. Acidic pH may favor development of certain cancers [oral, bladder; (137, 138) and, reversely, an alkaline pH may favor other cancers (cervical cancer; (139)].

#### 3.2.5 Bioelectricity: abnormal depolarization can promote cancer

Bioelectricity is another important component of the internal climate of the organism. The cellular resting potential is actively maintained by the activity of ion channels and pump proteins and is related to the differentiation state and proliferation of the cell (140, 141). Generally, a depolarized state is indicative of more undifferentiated cells (i.e., stem cells and cancer cells), while differentiation is associated with a more polarized state (142, 143). It has been proposed that abnormal depolarization of resting potential can be used as a marker for neoplasia, and depolarization may activate a metastatic phenotype in genetically-normal cells *in vivo* (144, 145).

#### 3.2.6 Biorhythm changes can lead to cancer

Many processes at the level of the organism are controlled through biorhythms (146). A change in the body biorhythm associated with night shifts (i.e., a global climate change induced by changes in life-style), for example, is known to lead to cancer development/progression (147, 148). Certain sudden changes in the organismic biorhythm parameters (through chemotherapy, for example) can also lead to cancer progression (149). In some cases, cancer cells lose the response to the body biorhythms and gain independence from the systemic clocks, which allows them to have constant access to the body nutritional resources (149).

#### 3.2.7 Basal metabolic rates affect cancer risk

Basal Metabolic Rate (BMR) is the number of calories burnt as the body performs basic (basal) life-sustaining functions. In a recent study, among normal weight participants, higher basal resting metabolism (BMR) was associated with elevated risks of esophageal adenocarcinoma and distal colon cancer among men; and proximal colon, pancreatic, thyroid, postmenopausal breast and endometrial cancers in women (150).

#### 3.2.8 Local and generalized inflammation can favor cancer

Both local and generalized systemic inflammation can promote cancer progression (cancer-promoting climate change); and cancer progression can also increase general levels of inflammation (cancer-induced climate change). Several factors can contribute to systemic inflammation. For instance, in addition to the many ways in which increased body fat level can affect health, the altered secretion of metabolically active, proinflammatory adipocytokines from adipose tissue is believed to play a key role in the mechanisms relating obesity and cancer, which are only starting to be uncovered (151). Moreover, obesity is associated with a state of chronic low-level inflammation, characterized by abnormal cytokine production which might affect both tumor initiation and tumor progression; for instance, adipocyte-conditioned medium can promote tumor migration (152, 153). On the other hand, tumor-induced circulating cytokines can be both used as climate markers and targeted for treatment purposes (154, 155). Epidemiologic studies suggest that in patients with several types of solid cancers, elevated circulating levels of C-reactive protein (CRP) are associated with poor prognosis, whereas in apparently healthy individuals from the general population, elevated levels of CRP are associated with increased future risk of cancer of any type (156).

The generalized inflammation associated with cancer progression may represent a physiologic process (i.e., a global climate change) that went out of control. Cancer becomes a sort of “auto-inflammatory” disorder like a “chronic shock”. Indeed, ultimately, in the terminal cases – when cachexia develops, cancer induces a kind of fatal “cytokine storm”. The excessive energy expenditure associated with prolonged inflammation may be a form of maladaptation (157).

## 4 Homeostasis and body climate control

The optimal functioning of an organism is ensured by homeostatic processes located at different levels (158). The first level involves automatic feedback mechanisms that control both cellular physiochemical processes and organ and tissular functions. The second level contains autonomous (self) regulation mechanisms trough which changes of the first level variables are sensed and adjusted (i.e., baroreceptor reflex). The third level is represented by neuron networks located in the central nervous system; this level integrates information transmitted from the second level with information from other sensory inputs from the outside world in order to coordinate the physiological and behavioral response to changing internal and/or external environmental conditions. A fourth level pertains to the bigger picture of human communities and social interactions that have a distinct influence on the organism. The key master-homeostatic coordinator that integrates all the different inputs and provides finely tuned responses maintaining the dynamic balance of the various properties of the organism is the hypothalamus.

Within the general homeostasis, the body also has specific systems that act as the body energy, weight and composition rheostats. The hormone leptin secreted by the adipose cells plays a critical role in whole body energy and weight homeostasis: lack of functional leptin or leptin receptors results in severe and early onset obesity in rodents and humans (159). Recently a leptin independent system has been demonstrated in rodents. This second system contains a sensor for body weight in the long bones of the lower extremities acting as “body scales”; and is part of a body weight homeostat, “gravitostat,” that keeps body weight and body fat mass constant (160). Besides these body weights rheostats, it also seems that body composition is tightly regulated. A minor decrease in liver glycogen increases the eating drive in order to replenish glycogen stores. By contrast, protein and fat imbalances are not tightly counter-regulated, leading to greater losses or gains in these individual components in response to nutrient intake (161). During fasting, liver glycogen shortage activates a liver–brain–adipose neural axis that has an important role in switching the fuel source from glycogen to triglycerides under prolonged fasting conditions (162). These rheostats are highjacked by cancer in order to direct the energy towards its growth.

**Figure 4:**
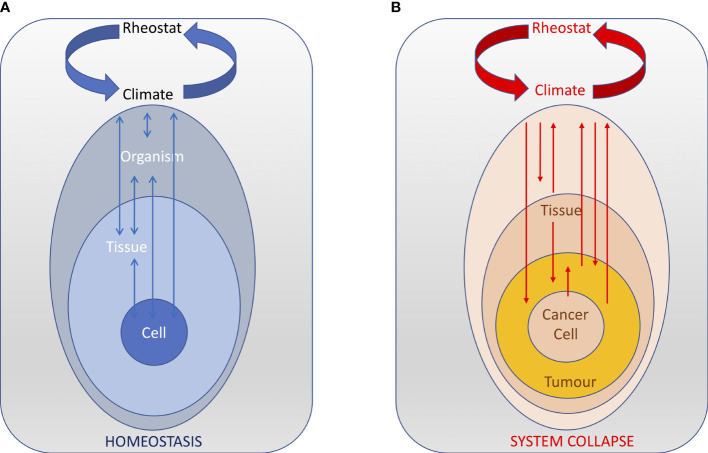
The central nervous acts as a rheostat that integrates all the body climate components that reflect the activities of, and the interactions among, all levels. **(A)** In normal conditions, the rheostat is able to ensure that the climate parameters remain in the functional range of the organism and the homeostasis of the system is maintained (arrows indicate stable reciprocal interactions between various levels and the climate). **(B)** During cancer progression, perturbations in various levels result in positive feedback loops that changes the body climate outside the range that the hypothalamus can control, ultimately resulting in the collapse of the system.

In our framework, the hypothalamus integrates all the climate components that reflect the activities of, and the interactions among, all levels to ensure the climate parameters remain in the functional range of the organism (**Figure 4A**). Internal climate variations induced by intrinsic and extrinsic factors, outside the range that the hypothalamus can operate, can result in permanent climate changes that, if not restored, can affect the proper functioning of the system and ultimately trigger its collapse. Some of these climate changes can promote cancer development and progression. But cancer itself can also induce irreversible climate changes that the hypothalamus cannot control anymore (**Figure 4B**). Alternatively, cancer-associated changes can directly affect the hypothalamus and its ability to maintain internal homeostasis, which ultimately results in a *permanent climate change*. Overall, we suggest that cancer is a disease of homeostasis – and its late stages reflect of the inability of the system to restore homeostatic control.

For instance, cytokines, like IL-1β and TNF-α, generated in the periphery during cancer progression are amplified and modified within the hypothalamus, leading to hypothalamic inflammation and aberrant activity of weight- and activity-modulating neurons that may induce muscle atrophy *via* activation of the hypothalamic-pituitary-adrenal axis (163, 164). Hypothalamic inflammation may be followed by dysregulation of homeostatic regulation of autonomic nerves (innervation of muscles, liver, fat tissue, endocrine glands and other organs) that may further potentiate dysregulation of metabolism and enhance peripheral, proinflammatory reactions (165). Hypothalamus appears to be an important contributor in the development and maintenance of the cachectic state (166).

## 5 Implications for prevention and treatment

Our framework, including the concept of an internal body climate that is the result of several intrinsic (including cancer) and extrinsic factors, and that – once perturbed, can affect many aspects of the body (including cancer development and progression) provides *a new perspective on cancer prevention and treatment*. Specifically, cancer prevention will encompass all the ways in which the internal climate can be maintained in the range that the hypothalamus can control, in response to both intrinsic and extrinsic factors. In terms of cancer management and treatment, we propose strategies that can slow down/stop climate change or reconstitute the climate by manipulating some of the climate components or modulate the rheostats.

### 5.1 Cancer prevention by maintaining a healthy internal climate and avoiding climate changes

Cancer prevention is often discussed in terms of life-style choice that diminish the potential for mutations, by avoiding sun exposure, smoking and other chemical mutagens. Our framework emphasizes more general life-style changes that affect the internal body climate in two directions: (i) to promote a climate that can mitigate the direct effects of mutagens (i.e., in terms of reducing oxidative stress and inflammation) and (ii) to avoid climate changes that can promote cancer development and progression.

In our view, besides hereditary cancers that represent a minority, at least 90% of cancers are ultimately caused by internal climate changes. These include both cancers initially triggered by external climate factors (e.g., small cell lung cancer related to cigarette smoking, or cervical adenocarcinoma caused by a human papilloma virus infection) and cancers promoted by internal climate changes (e.g., breast cancer in women and prostate cancer in men related to hormone levels). Both the external and the internal body climate influences on the organism`s genome have been characterized (167, 168). Mutations induced by carcinogens, radiation, or endogenous sources may promote, inhibit or have neutral consequences for cancer inception and progression (169–171). But the internal body climate can modulate the effect of these mutations and direct the cancer trajectory towards progression or extinction.

For instance, it is important to note that even for cancers in which the external environment plays an important role, the internal climate’s participation is crucial in order for mutated cells to progress to malignancy as only a minority of patients exposed to different noxa, carcinogens, viruses, radiation, etc, develop cancers. Free radicals produced by substances present in cigarettes, sun exposure, and some chemicals can induce oxidative DNA damage that could lead to cancer. But anti-oxidants produced by our bodies or acquired from our diets work by preventing such oxidative damage and thus have protective effects against cancer.

Nevertheless, when cancer is fully developed, anti-oxidants may also protect cancer cells and may accelerate cancer progression (172, 173). Interestingly, the source of anti-oxidants appears to be a key factor. For example, a high *dietary* intake of beta-carotene is associated with a modest decrease of lung cancer incidence (174), but, among smokers, the use of beta-carotene *supplements* actually appeared to increase the risk of lung cancer (175). Similarly, dietary vitamin E is associated with a lower risk of developing prostate cancer, but a study evaluating vitamin E supplements found an increased risk (176). Also, another study demonstrated that end-stage patients with cancer who had a predicted life expectancy of only 12 months had a median increase in survival of five months when treated with a supplement of coenzyme Q10, vitamins A, C, and E, selenium, folic acid, and beta-carotene (for those without lung cancer) (177).

Diet can influence the climate – and the potential for cancer development and progression, in many other ways (178). Interestingly, caloric restriction – by decreasing the food intake by 30-40%, has been shown across multiple studies to significantly inhibit the growth of diverse tumor types, including breast, lung, prostate, brain, pancreatic and colorectal cancer [reviewed in (178)]. Specific components of the diet can also have an effect on the internal climate, and – directly or indirectly, on cancer.

For instance, regarding protein consumption, a study of more than 6,000 subjects reported that 50-65 year-old subjects who ate a high-protein diet had a four-time higher risk of developing cancer compared to those who had a low-protein diet. Interestingly, above age 66, subjects on a low protein diet had an increased cancer risk. In this age group, subjects with high protein consumption had a 60% reduction in cancer mortality (179).

Data regarding the role of cholesterol in cancer is also controversial. A large metanalysis did not find any role of lowering blood cholesterol for cancer prevention (180). At almost the same time, another large case–control study with 295,925 cancer patients, suggested a link between statin use and a slight reduction in cancer-related mortality for 13 different cancer types, including prostate, colon, lung and urinary (181). This difference may be due to the heterogeneity of lipid metabolism in cancer. In different types of cancers, different sets of cholesterol synthesis enzymes are either upregulated or downregulated (182) and before using specific targeted inhibitors their levels should be determined. For example, ATP-binding cassette transporter A-1 (ABCA1) is upregulated in melanoma but downregulated in colon cancer (182). It has been suggested that in some cancer types, like pancreatic cancer for example, combining inhibitors of low-density lipoprotein receptor (LDLR), which facilitates cholesterol uptake, and chemotherapy leads to a synergistic anti-tumor effect (183).

Fatty acids have been classically associated with cancer growth and progression. They sustain membrane biosynthesis during rapid proliferation, and are an important energy source for cancer cells during conditions of metabolic stress (184). Omega-6 (n-6) polyunsaturated fatty acids (PUFA) (e.g., arachidonic acid (AA)) and omega-3 (n-3) PUFA (e.g., eicosapentaenoic acid (EPA)) are precursors to potent lipid mediator signaling molecules, termed “eicosanoids,” which have important roles in the regulation of inflammation. In general, eicosanoids derived from n-6 PUFA are proinflammatory while eicosanoids derived from n-3 PUFA are anti-inflammatory (185). An extensive systematic review assessing the relationship between PUFAs consumption and cancer risk found increasing total PUFA may very slightly increase cancer risk, offset by small protective effects on cardiovascular diseases (186). On the other hand, it has been previously shown that a low dietary ratio of omega-6 to omega-3 fatty acids may delay progression of prostate cancer (187). Also, dietary lipids can promote metastasis through fatty acids interacting with the fatty-acid receptor CD36 on metastasis-initiating cells (188). Furthermore, it has been recently shown that dietary palmitic acid (PA), but not oleic acid or linoleic acid, promotes metastasis in oral carcinomas and melanoma in mice (189).

The evidence of an association between carbohydrates consumption and cancer risk is scanty. There is some data for an association between fiber and colorectal cancer, where increased consumption is associated with reduced risk of disease development and mortality after diagnosis (190). There is also limited data suggesting an increased risk of colorectal cancer associated with high intakes of sucrose and an increased risk of ovarian cancer associated with high intakes of lactose (191). Different susceptibilities to colorectal cancer have also been correlated with dietary differences that reflect in different compositions of gut microbiota (192).

Other interventions – such as anti-inflammatory diets, appropriate rest, stress reduction and exercise, can strengthen the immune system, which in turn can contribute to cancer prevention. Systemic inflammation can be manipulated through dietary interventions. Host metabolism and energy balance are influenced by an interplay between the intestinal microbiota, bile acids and nutrients that may impact global inflammation, immune responses, gut hormone secretion and neuronal activity (193). Systemic changes associated with exercise can also have a beneficial effect on cancer prevention and has been demonstrated to reduce breast cancer recurrence (194). The positive impact of exercise on the immune function (195) and its impact on decreasing the incidence of breast cancer has been well documented (196–199). Similar effects have been described for several other cancer types (200): hematological malignancies (201), bladder cancer (202), colon cancer (203), esophageal cancer (204), stomach cancer (205), kidney cancer (206) and endometrial cancer (207). For lung cancer, a meta-analysis of 25 observational studies (208) demonstrated that physical activity is associated with reduced risk of lung cancer among former and current smokers but not among never smokers. For cancers of the pancreas, prostate, ovaries, thyroid, liver, and rectum, the impact of physical activity for cancer prevention is not as clear (209, 210). Besides improving the immune function, there are several mechanisms that may explain the impact of physical activity on cancer (211), including helping to prevent and treat obesity that is a risk factor for many cancers, reducing inflammation and preventing high levels of insulin, which has been linked to breast and colon cancer development (212).

### 5.2 Cancer treatment through internal climate interventions

Most oncology treatments available today are directed towards cellular (chemotherapy and targeted agents) and tissular (angiogenesis inhibitors and immunotherapy) levels. Developing new interventions directed towards affecting the organism’s internal climate should also be developed. In our framework, cancer is associated with internal climate changes that sustain cancer development and progression, but cancer itself can also induce climate changes that affect the general health status and homeostasis of the organism. Thus, manipulating the internal climate can have two effects: (i) slow or stop cancer progression and (ii) restore homeostasis or improve the general health of the patient. What type of changes can be induced in the body climate in order to decrease local cancer progression and the development of distal metastases?

Two approaches can be used:

A. Manipulate key climate components to restore climate to levels that do not support cancer progression
B. Modulate the function of the master-homeostat

The key for using the internal climate framework for individual cancer types is to define the internal climate factors and components that can be manipulated in a particular clinical setting in order to prevent cancer progression. Note that in addition to affecting cancer progression, the internal climate can also affect the efficacy of treatments. For instance, as mentioned earlier, commensal bacteria can promote endocrine resistance in prostate cancer through androgen biosynthesis (48), and some microbiota species can interfere with the response to immunotherapies (see below). At the opposite spectrum, fasting appears to decrease the cytotoxic effects of chemotherapy on normal cells (discussed next). While some of the body climate components that could be manipulated for treatment purposes are the same as those discussed for prevention, the types of changes required to restore a climate already affected by cancer are different. Below we provide an overview of such internal climate manipulation strategies.

#### 5.2.1 Manipulating the internal climate for treatment purposes

##### 5.2.1.1 Intermittent fasting and diet changes can induce climate changes with negative effects on cancer progression

Intermittent fasting has been proposed as a means to decrease tumor burden as well as the negative effect of chemotherapy (213). For instance, in several mouse models intermittent fasting decreased the rate of metastasis, prolonged survival, and reduced tumor growth, as measured by weight or volume (214). Although some of these studies are controversial and suggest potential detrimental effects in certain oncologic conditions [and the human data is currently scarce (215)], preliminary studies suggest that prolonged fasting in some patients who have cancer is safe and potentially capable of decreasing chemotherapy-related toxicity and tumor growth (216).

Cancer dependency on certain aminoacids may also be utilized for therapeutic purposes. A recent review described different diets that can impact cancer development (217). In a mouse model of breast cancer, asparagine restriction reduced the metastasis of breast cancer without affecting the growth of the primary tumour (218). However, asparagine restriction is difficult to implement in human patients. Other amino acid depleted diet approaches may by more feasible for patients. Serine depletion for example, either by removing it from the diet, or by enzymatic inhibition may have a therapeutic benefit especially in tumors with TP53 mutations (219). Recently, the therapeutic benefit of combining serine synthesis inhibition with dietary restriction of serine and glycine was demonstrated in an animal model (220).

Interesting, several *in vitro* studies suggested that water with a low deuterium concentration (<65 ppm) may inhibit cancer growth (221–229) and augments the inhibitory effect of paclitaxel (230). Anecdotal reports and clinical data from Europe suggest that lowering the levels of deuterium in the body by drinking deuterium depleted water (10-20 ppm) may improve quality of life and possibly survival of cancer patients (231–233).

##### 5.2.1.2 Climate reconstitution though microbiota manipulation

Recently, microbiota has drawn a lot of attention as probiotics, prebiotics and synbiotics (a mixture of prebiotics and probiotics) have demonstrated putative beneficial effects in the treatment of cancer (234). Enrichment in some species has been associated with response to PD-1 and CTLA-4 blockade in humans, while other species have been negatively associated with response to anti-PD-1 and anti-CTLA-4 therapy (235). As pointed out by Gong et al. (235), at present, these results are inconsistent between different research groups and they are not yet ready for clinical use. Studies using fecal transplant for improving response to immunotherapy are currently in development (236).

##### 5.2.1.3 Biorhythm modulation and chrono-immunotherapy can slow cancer progression

In several mouse models, circadian disruption was shown to accelerate cancer progression, whereas circadian reinforcement/reprogramming slows it down. The reinforcement of the host circadian timing system with fixed meal times induced 24-hour rhythmic expression of critical genes in clock-deficient tumors, which translated into cancer growth inhibition (237). A decade later, a similar experiment confirmed these earlier findings (238). The circadian rhythm of the immune cells has been described in several studies, with the number of circulating CD4T and CD8T cells near doubling in the evening compared with morning (239–242). Lately, it has been demonstrated that more frequent morning or early afternoon administration of immunotherapy nearly doubled overall survival as compared to more frequent late afternoon or early evening treatment administration (243). More recently, the finding that key circadian rhythm hormones such as melatonin, testosterone and glucocorticoids influence CTC generation dynamics (with most spontaneous CTC intravasation events occurring during sleep) provides a new rationale for time-controlled treatment of metastasis-prone cancers (244).

##### 5.2.1.4 Internal body pressure manipulation can improve survival of cancer patients

An easy way to manipulate whole body pressure is by adjusting blood pressure. Beta-blockers, for example, like propranolol, that decrease blood pressure alter the metastatic potential of cancer cells (245). Melanoma patients who received immunotherapy while taking pan β-blockers lived longer than patients who received immunotherapy alone or patients that received immunotherapy and β1-selective blockers (246). Bisoprolol is another selective β1-blocker commonly used to treat hypertension, cardiac ischemia, and congestive heart failure. Bisoprolol improved survival, increased total heart mass, and other heart parameters and, importantly, improved food intake and activity levels in an AH-130 tumor-bearing rat models (247).

##### 5.2.1.5 Increasing whole body temperature has a benefic anti-cancer effect

In human patients, regional and whole-body hyperthermia in combination with radiotherapy and/or chemotherapy, has been shown to significantly improve therapeutic outcomes in more than sixty clinical trials (248). Increasing regional and whole-body temperature has been acknowledged by the American Cancer Society as a potential cancer treatment modality. Cancer regression associated with fever has been reported for more than 150 years (249). Pre-clinical studies showed a benefic effect of hyperthermia on immune cells, and, recently hyperthermia showed benefit in combination with check-point inhibitors in two dozen animal models (250, 251).

##### 5.2.1.6 Restoration of internal pH may inhibit metastasis

Although dietary acidosis alone is not sufficient to increase cancer risk (252), various alkalinization methods of pHe have been proposed as cancer treatment for more than six decades (253) with limited success (254). Initial studies done in mouse models showed that the administration of alkaline or buffering agents did not decrease the growth of primary tumors (255) but may inhibit metastasis (256). However, the effectiveness of systemic manipulation of pHe through ingesting certain alkaline products is controversial as blood pH buffering systems are very robust in maintaining the pH at a physiologic value of 7.35.to 7.45 (257). On the other hand, studies targeting directly acid-base transporters and proton-sensing receptors that permit cancer cells to sense and adapt to the acidic tumor microenvironment are currently in development (133). Therapeutic benefits of chemo- or targeted therapy in combination with an alkaline diet – defined as more vegetables and less meat, on the survival of patients with lung and pancreatic cancer have also been reported (258–260). Furthermore, drugs, like Veverimer, that effectively decrease systemic acidosis are interesting candidates, but their benefit has not yet tested in cancer patients (261).

##### 5.2.1.7 Modulating bioelectricity to treat cancer

The idea of using electricity to treat cancer has led to some interesting results. First, Tumor-Treating Fields (TTFields) are low intensity, intermediate frequency, alternating electric fields delivered externally through noninvasive transducers placed locoregionally around the anatomic region of the tumor (262). TTF have been FDA-approved for the treatment of brain cancer (263) and mesothelioma (264). Also, a device using tumor-specific amplitude-modulated radiofrequency electromagnetic fields (AM RF EMF) has been also approved in Europe since 2018 for the treatment of liver cancer (265). Several proposals have been previously made to consider cancer a “channelopathy” (266) and the modulation of the bioelectricity of cancer cells using targeted agents directed to the membrane ion channels, as suggested by Levin (267), may represent a novel way of cancer treatment. Interestingly, both TTF and AM RF EMF treatments work by activating specific calcium channels and result in increased levels of cytosolic Ca^2+^ (265). Thus, it is plausible that similar effects can be obtained by directly manipulating cancer cell calcium membrane channels.

Targeting cancer’s ion channels is an attractive therapeutic option (268), and, in a previous large cohort study of 66,806 men and women observed longitudinally for 6 years, every 100 mg per day decrement in magnesium intake was associated with a 24% increase in the incidence of pancreatic cancer (108). One of the main functions of magnesium in the human body is the maintenance of cellular ionic gradients, keeping intracellular sodium and calcium low and potassium high.

##### 5.2.1.8 Body weight loss can slowdown cancer progression

There is clear evidence that obesity increases the risk and worsens the prognosis of many common cancers. Thus, weight management is crucial to patients with cancer and cancer survivors (216). A recent study in both mouse models and humans has shown that, although obesity boosts the metastasis of breast cancer cells to the lung by recruiting neutrophils to the lung pre-metastatic niche, weight loss is sufficient to reverse this effect (269).

##### 5.2.1.9 Manipulating oxygen levels might be beneficial

Although hypoxic microenvironments can promote tumor growth, according to the U.S. Food and Drug Administration, there is no evidence that hyperbaric oxygen treatment is effective in treating cancer. In fact, quite the opposite has been noticed: a decline in the oxygen pressure was associated with a decline in the risk of cancer (270). Anecdotally, in the clinical experience of one of us, we noted that oxygen supplementation given intermittently under pressure at night may be associated with a clinical benefit. Specifically, we have been following a patient diagnosed with extra-skeletal chondrosarcoma with multiple bilateral lung metastasis for more than 4 years (2012–2016). At the time of presentation in 2012 the patient was also on a Continuous Positive Airway Pressure CPAP machine that he has been using to improve his symptoms of sleep apnea. Despite a very high tumor burden present in both lungs, his performance status remained unchanged for four years.

##### 5.2.1.10 Increasing carbon monoxide levels can have multiple anti-cancer effects

Intriguingly, recent data shows that low dose of carbon monoxide administered systemically may inhibit cancer metastasis. It has been demonstrated in several pre-clinical studies that carbon monoxide-releasing molecules have anti-proliferative, pro-apoptotic, anti-angiogenic and anti-metastatic properties and may be exploited in the near future as non-conventional cancer treatment agents (271).

##### 5.2.1.11 Improving the systemic immune function through life-style changes

Immune function may be improved by life-style changes that affect the internal climate, such as exercise, diet manipulation, better sleep, relaxation techniques; most of which of also have a preventative role (discussed above). For instance, exercise has been long been associated with improvements in clinical, functional, and, even, survival outcomes in patients with breast, colorectal, and prostate cancers (130, 272, 273). Exercise continues to have a positive effect on survival even in terminal forms of cancer (274, 275). The immune function is modulated by diet and the influence of various diet components on cancer growth and response to immunotherapy has been described but a detailed characterization of the impact of different diet components on cancer development is presently lacking (276, 277). Several studies demonstrated a positive relationship between a restful sleep and the immune system (278). A classical review failed to demonstrate a clear benefit of meditation in improving the immune system function (279). However, more recently, a carefully designed study done with more than a hundred subjects demonstrated a robust activation of the immune system following an intense 8-day meditation retreat (280). Stress has long been shown in several studies to negatively influence cancer survival. A new study identifies a stress-induced response in dendritic cells – the activation of the glucocorticoid-inducible transcriptional regulator TSC22D3 as a potent, immunosuppressive effect of stress on cancer (281). Studies in various cancers have shown that patients taking a beta-blocker have higher survival and lower recurrence and metastasis rates (282).

##### 5.2.1.12 Rejuvenating the internal climate through senolytics

80% of cancers occurs after age 55 (283). Older age is associated with significant changes in both cancer, microenvironemt and body climate (284). Interestingly, colorectal cancer incidence increases with age whereas metastatic spread declines (285), which points to the possibility that different biological factors may induce cancer growth and metastasis. The presence of senescent cells in the tumoral microenvironment has been recently acknowledged as a key hallmark of cancer (286); and it is likely that senescent cells contribute to climate aging, a key risk factor for cancer development. In the near future, cancer patients may benefit from treatments targeting tumor-promoting senescent cells, either by pharmacologic or immunologic ablation of these cells (287). Studies using senolytic combinations have been completed in mice and are currently on-going in humans (288, 289).

##### 5.2.1.13 Reducing co-morbidities can increase patient survival

Systemic co-morbidities have been found to be associated with both cancer initiation and progression (290) in a significant number of patients (>50% in some series) (291, 292). Co-morbidities represent an unappreciated source of body climate alterations and their appropriate management may improve the survival of cancer patients. In one series, for example, the most common co-morbidities associated with cancers of the lung, colon, rectum and Hodgkin lymphoma were diabetes, COPD and hypertension. The clinical course of these three co-morbidities may be improved through a concerted management of a multidisciplinary team of medical specialists (293). The relevance of the body climate is also underscored by the incidence of secondary malignancies that has been increasing steadily over the last two decades and occurs now in approximately one in five cancer patients (294).

#### 5.2.1 Hypothalamus-related targeted interventions

In the context of systemic inflammation, the hypothalamus integrates signals from peripheral systems, translating them into neuroendocrine perturbations, altered neuronal signaling, and global metabolic derangements (295). Cancer is a homeostatic challenge to the organism (296, 297), and, specifically, neural circuitry is disrupted in cancer (298). Decline in the body`s homeostatic mechanisms are associated with cancer progression, and in turn, cancer progression affects body homeostatic mechanisms. In the terminal phases of cancer, the hypothalamic inability to maintain homeostasis at the organism level triggers the cascade of systemic events associated with cachexia. Systemic biomarkers that sense climate changes can be used both for early detection and disease monitoring as well as potential hypothalamus-related interventions.

For instance, serum from animals held in an enriched environment (EE) defined as “a combination of complex inanimate and social stimulation” (299) inhibited cancer proliferation *in vitro* and was markedly lower in leptin (300). Hypothalamic brain derived neurotrophic factor (BDNF) was selectively upregulated by EE; its genetic overexpression reduced tumor burden, whereas BDNF knockdown blocked the effect of EE. The hypothalamic BDNF downregulated leptin production in adipocytes *via* sympathoneural β-adrenergic signaling (300).

In addition to the well-known association between aging and cancer, an intriguing observation links hypothalamus to aging (301). We can speculate that rejuvenation of hypothalamic function through targeted approaches such as use of microelements like chromium-picolinate (301) may also be beneficial for cancer patients.

## 6 Perspectives

As mentioned at the onset, the goal of our approach is to shift the focus from exploring changes at the DNA, cell or tissue levels to investigating changes that affect the entire body climate and use our understanding of these changes to both prevent and predict cancer development as well as slowdown cancer progression. To do so, we need to (i) identify relevant ‘*body climate factors and components*’ that affect and are affected by cancer, (ii) develop ‘*body climate biomarkers*’ that apply to either specific or more general cancer types, (iii) use these biomarkers to define ‘*body generalized and personalized climate scores*’ that would allow us to predict the likelihood of cancer development and intervene, and (iv) develop strategies to *prevent ‘body climate changes’*, stop or slow the changes, or even revert the changes.

Here, we briefly overviewed the many factors that affect the multitude of internal climate components and the interactions among them, in the context of cancer promotion, prevention, and treatment. Although we believe the available information supports the role of the internal climate in promoting as well as preventing and treating cancer, the current data is yet to be systematically reviewed and analyzed. We hope that such endeavors will prompt proper experimental studies to define the main climate components that are most associated with cancer. Such studies will also allow for the development of systemic climate biomarkers. Once such biomarkers are identified, thorough meta-analyses of parameter values and other physiological aspects should allow the development of cancer climate scores. This approach is similar to that used by Akinyemiju et al. (302) to define the so-called “allostatic load score”.

Allostasis has been defined by Romero et al. as the active process of maintaining/re-establishing homeostasis (303). The term allostatic load (AL) has been introduced in order to describe the physiological burden of cumulative stress on biological systems normally involved in acclimation to environmental challenges. The allostatic load score (used to quantify the allostatic load) was defined as the sum score of the number of biomarkers above a set threshold (302). These biomarkers include: serum albumin < 3.8 g/dL, C-reactive protein (CRP) > 3 mg/L, high-density lipoprotein (HDL) < 40 mg/dL, total cholesterol >240 mg/dL, heart rate >90 beats/min, systolic blood pressure > 140 mmHg, diastolic blood pressure >90 mmHg, serum creatinine >1.3 mg/dL, and blood urea nitrogen (BUN) >18 mg/dL, waist circumference (WC) > 88 cm in females and >102 cm in men (302). Interesting, for cancer mortality, every unit increase in AL score increased the risk by 17% among those with normal BMI and by 9% among those who were overweight/obese. A separate mini meta-analysis found that a one-unit increase in AL score was associated with a 9% increased risk of cancer-specific mortality (304).

Significant for the internal climate perspective introduced here, the AL score biomarkers are all related to the climate components. In order to better quantify the impact of different cancer types on the climate components, a general cancer climate score can be designed that would include besides the 10 AL biomarkers, other general biomarkers like Neutrophil/lymphocyte ratio, plasma levels of ctDNA, insulin like growth factor (IGF1), growth hormone (hGH) and methyl malonic acid (MMA), for example. This general climate score can be further refined to include specific biomarkers relevant for individual cancer types type i.e. (specific metabolites, specific exosomes, etc). The general and the personalized climate scores may guide different cancer therapies and monitor recurrence and, also, be used as prognostic tools. As an example, an increase in methylmalonic acid levels may be indicative of increased risk of metastasis (305).

In a thought-provoking study, a machine-learning algorithm that integrated blood parameters, dietary habits, anthropometrics, physical activity, and gut microbiota measured from a cohort of patients accurately predicted personalized postprandial glycemic responses to real-life meals. A blinded randomized controlled dietary intervention based on this algorithm resulted in significantly lower postprandial responses and consistent alterations to gut microbiota configuration (306). A similar approach can be effective for cancer patients in whom parameters like ctDNA, methylated DNA, glycoproteins, metabolites, exosomes or other biomarkers obtained through liquid biopsies can guide a nutritional intervention program.

Notably, Earth weather changes can be predicted only within a limited time frame (two weeks or so) (307); and Earth climate changes are grossly unpredictable. One of our central hypotheses is that the climate factors favorable to malignant transformation and progression follow a deterministic dynamic, such that cancer can be predicted long before its clinical appearance. This would allow the implementation of changes that may avoid cancer development. Such incipient climate disturbances that promote oncogenic changes may involve the immune system, the systemic metabolism, both cellular and whole-body biorhythms, and neuro-endocrine disturbances. Specific climate biomarkers can be used to predict the inception of different cancer types in preclinical stages and also to monitor cancer progression, treatment responses and the risk of metastases before clinical metastases develop. Candidate biomarkers that can identify cancer at a pre-clinical stage and monitor disease progression include metabolites, exosomes and glycoproteins. For instance, an increase in the serum level of branched chain amino acids is thought to precede with several years the development of pancreatic cancer (58, 59).

## 7 Conclusion

All four enabling characteristics of the hallmarks of cancers (genome instability, tumor promoting inflammation, non-mutational epigenetic reprogramming and polymorphic microbiomes) described in three consecutive papers (56, 308) can be modulated by the internal climate of the body. Genome instability is also considered one of the hallmarks of aging (309) and most components of body’s internal climate are significantly altered in elderly persons. Systemic inflammation has been associated with obesity (310), changes in blood concentration of different microelements (311), stress (312) and gut microbiome composition (313). Both DNA methylation and histone modifications are vulnerable to disruption by endocrine disruptive compounds exposures (314). In terms of the influence of internal body climate on microbiota, fever-dependent shifts in the gut microbiota were recently demonstrated (315), so fever may influence cancer progression either directly by influencing cancer growth rates or indirectly by stimulating the function of immune cells or by selecting for a certain microbiota composition.

The internal body climate components are co-dependent and, in individual patients, their collusion creates a perfect storm that leads to a cancer-prone environment. Age is the key altering factor of internal body climate and, given the causal relation between aging and cancer, although the cancer incidence related to external factors may be reduced through lifestyle changes, the incidence of age-related cancers will likely continue to increase in the near future alongside with the increase of the human population life span. A recent report (316) described the shift to cancer as the leading cause of death in the highest-income counties in the United States of America, surpassing cardiovascular diseases. This trend is also present in several high income and high-middle income countries (317).

In this paper, we propose that the internal body climate can directly or indirectly influence all cancer hallmarks and represents an under-appreciated element in cancer prevention and treatment. Importantly, the different climate components and factors described here can be used to generate climate scores and guide body climate-directed interventions. Integrating body climate interventions with the existent therapeutic modalities may represent the cancer treatment paradigm of the immediate future that will impact on cancer patients’ quality of life and survival.

## Data availability statement

The original contributions presented in the study are included in the article/supplementary material. Further inquiries can be directed to the corresponding author.

## Author contributions

All authors listed have made a substantial, direct, and intellectual contribution to the work and approved it for publication.
