# Supplementary material for: The underexplored links between cancer and the internal body climate: Implications for cancer prevention and treatment
Source: Front Oncol. 2022 Dec 22;12:1040034. doi: 10.3389/fonc.2022.1040034 (PMC9815514; doi:10.3389/fonc.2022.1040034)
Supplement: Supplementary file 1 [file DataSheet_1.docx]

**Supplementary Material**

1. **The impact of hormones and other active molecules on cancer development**

The levels of circulating hormones are also good indicators of health and internal climate status. Changes in the levels of many hormones have been demonstrated both in animal models and in large epidemiological studies to play a key role in the development of many types of cancer, including breast, prostate, uterine, ovarian, testicular, thyroid and bone cancers (1-20)

For instance, hyperthyroidism is associated with breast cancer risk (8). On the other hand, hypothyroidism has been thought to reduce cancer aggressivity (9); however, a meta-analysis has shown that while it was associated with a decreased risk of prostate cancer, it may be associated with an increased risk of colorectal cancer and hepatocellular carcinoma (10). High levels of insulin have also been associated with an increased risk of breast, endometrial, ovarian and prostate cancer, increased breast and pancreatic cancer mortality and also increased of any cancer mortality. This association holds true regardless of the body habitus of the patient (normal weight vs obese) (17).

Adrenergic neurotransmitters such as epinephrine or norepinephrine can be released by the adrenals into the bloodstream in support of the ﬁght-or-ﬂight reﬂex in response to the activation of the sympathetic nervous system (SNS) through the sympathetic - adrenal - medullary axis. The release of these neurotransmitters has been linked to tumor development associated with stress (18), and thus can be considered a cancer-promoting body climate change induced by external factors. Catecholamine-mediated suppression of cellular immunity may also play a role in increased growth of certain tumors (19). Recent data suggest that stress hormones, particularly glucocorticoids, may be associated with cancer progression and can negatively influence cancer treatment (20). Many other hormones can also induce tumor growth and metastasis (21).

The role of estrogen in promoting the progression of ER^+^ breast tumors has been well described and, recently, it has been shown that women with a history of breast cancer taking systemic hormonal replacement therapies are at a high risk of recurrent disease (11). However, the role of estrogen in promoting the growth of ER^−^ breast cancers has been generally overlooked (12). For instance, in a mouse model it has been found that estradiol also promotes brain metastasis of ER^−^ breast cancer cells by modulating astrocyte function. Thus, endocrine therapies (i.e., a change in a cancer-promoting climate component) used in the treatment of ER^+^ breast tumors may provide some clinical benefit towards reducing and managing brain metastases in patients with ER^−^ breast tumors (13).

Interestingly it is possible that the impact of estrogen on tumor growth is mediated by myeloid cells. The indirect role of estrogens in tumor growth has been recently demonstrated in a melanoma model. Estrogens modify the macrophage phenotype toward an immune-suppressive type that promotes CD8^+^ T cell dysfunction and exhaustion and resistance to check point inhibition (14). These findings are consistent with the observation that the response to checkpoint inhibitors in men is better than in women (22) but recently this observation has been readdressed (23).

Endocrine therapies are also used in the treatment of prostate cancer. However, for prostate cancer there is an inverse association between tumor aggressivity and testosterone levels, with lower levels of testosterone being associated with more aggressive disease (16,17). It has been speculated that low serum testosterone may lead to activation of oncogenic pathways but the details of this mechanism are unclear.

Other hormones, like melatonin, for example, may inhibit cancer metastasis (24). Patient with cancer have poor sleep and this may influence melatonin secretion. In a recent study, serum melatonin levels in women with breast cancer correlated significantly with self-reported sleep quality and psychometric profiles of depression (25).

1. **The impact of microelements on cancer progression and prevention**

Microelements, although found in extremely low levels, are an important internal climate component that can play complex roles in cancer (i.e., act as cancer-promoting or cancer-preventing climate components); as well as can be affected by cancer. Low levels of **zinc** have been related to an increase incidence of several types of cancer (26). On the other hand, high levels of zinc have been associated with a higher risk of lymph node metastasis in thyroid cancer (27). An increased level of **selenium** (28) was found to be related to a lower risk of breast cancer but another study did not find any relation between increased selenium uptake and cancer risk (29). Increase of **phosphorus** intake may be related to cancer but on the other hand hypophosphatemia is a common occurrence in cancer patients and can be induced by different agents (30,31).

Initially, it has been thought that excess **iron** can contribute to tumor initiation and tumor growth; for instance, high intake of dietary iron is associated with an increased risk for some cancers, particularly colorectal cancer (32). More recently, it has been shown that reduced iron intake and low systemic iron levels are also associated with the pathogenesis of colorectal cancer, suggesting that iron intake must be carefully balanced in order to ingest an optimal dose that will avoid both iron deficiency and iron excess (i.e., iron might be an important climate component affecting cancer risk) (33,34). Also, the source of iron seems to play a role, as intake of iron from plants or from white meat were found to be inversely associated with the risk of colorectal cancer, while iron from red meat was found to be positively associated with colorectal cancer risk (35).

Similarly, for more than a decade it has been known that **magnesium** may have pleiotropic, often opposing effects on tumor growth, vascularization, and metastatic potential, such that both favorable and unfavorable effects have been associated with this oligomineral (36). Epidemiological studies have suggested that magnesium deficiency is a risk factor for several types of human cancers including colorectal and pancreatic cancer (37-39).

Several retrospective and prospective studies demonstrated that **calcium** may provide some protection against colorectal cancer, but, at present, the evidence of using calcium supplements is inconsistent. Therefore, at present, NCI does not recommend the use of calcium supplements to reduce the risk of colorectal or any other type of cancer. On the other hand, a prospective study investigating dairy product and calcium intakes among more than 29,000 men participating in the National Cancer Institute’s (NCI) Prostate, Lung, Colorectal, and Ovarian (PLCO) Cancer Screening Trial showed increased risks for prostate cancer associated with high dietary intakes of calcium and dairy products, particularly low-fat dairy products. Interestingly, calcium from supplements was not associated with increased prostate cancer risk (40-47).

Experimental studies demonstrated the co-carcinogenic effect of **salt** through synergic action with *Helicobacter pylori* infection (48). A systematic review of prospective population studies clearly demonstrated an association between salt consumption and the incidence of gastric cancer (49).

*In vitro* data suggested a potential suppressive effect of **manganese** on prostate cancer. It has been also showed recently in a mouse model that manganese is essential in innate immune sensing of tumors and enhances adaptive immune responses against tumors (50). Importantly, the authors conducted a phase 1 study and demonstrated the synergistic effect between PD-1 inhibition and manganese administration (51).

Similarly, decreasing **copper** levels may reduce mitochondrial production of energy in cancer cells leading to decreased metastasis in a triple negative breast cancer mouse model (52). Interestingly, the growth of the primary tumors was not affected by copper depletion. The drug used to lower the systemic levels of copper is called tetrathiomolybdate. This drug also disrupts the collagen in the metastatic niche making it more difficult for distal metastasis to form (53)

Several extrathyroidal malignancies, including breast and prostate, express the sodium/iodine symporter (54), and it has postulated that **iodine** deficiency may be associated with increased risk of some cancers (55,56). Recently, a high level of serum iodine combined with a high level of serum selenium was found to be associated with a lower risk of breast cancer (57).

**References**

1. Dall GV, Britt KL. Estrogen Effects on the Mammary Gland in Early and Late Life and Breast Cancer Risk. Front Oncol. (2017) 7:110.

2.Daniels NA, Nielson CM, Hoffman AR, Bauer DC Osteoporotic Fractures In Men (MrOS) Study Group. Sex hormones and the risk of incident prostate cancer. Urology. (2010) 76(5):1034-40.

3.Kaaks, R., Lukanova, A. & Kurzer, M. S. Obesity, endogenous hormones, and endometrial cancer risk: a synthetic review. Cancer Epidemiol Biomarkers Prev (2002) 11, 1531–1543.

4.Ho SM. Estrogen, progesterone and epithelial ovarian cancer. Reprod Biol Endocrinol. (2003) 1:73.

5. De Toni L, Šabovic I, Cosci I, Ghezzi M, Foresta C, Garolla A. Testicular Cancer: Genes, Environment, Hormones. Front Endocrinol (Lausanne). (2019) 10:408.

6. Huang H, Rusiecki J, Zhao N, Chen Y, Ma S, Yu H, et al. Thyroid-Stimulating Hormone, Thyroid Hormones, and Risk of Papillary Thyroid Cancer: A Nested Case-Control Study. Cancer Epidemiol Biomarkers Prev. (2017) 26(8):1209-1218.

7. Poidvin A, Carel JC, Ecosse E, Levy D, Michon J, Coste J.. Increased risk of bone tumors after growth hormone treatment in childhood: A population-based cohort study in France. Cancer Med. (2018) 7(7):3465–73.

8. Yang H, Holowko N, Grassmann F, Eriksson M, Hall P, Czene K. Hyperthyroidism is associated with breast cancer risk and mammographic and genetic risk predictors. BMC Med. (2020) 18(1):225.

9. Krashin E, Piekiełko-Witkowska A, Ellis M, Ashur-Fabian O. Thyroid Hormones and Cancer: A Comprehensive Review of Preclinical and Clinical Studies. Front Endocrinol (Lausanne). (2019) 10:59.

10. Trodello C, Higgins S, Omeed A, Wysong A Hypothyroidism as a Risk Factor for Cancer: A Systematic Implications for Future Studies. Cancer Sci Res Open Access (2017) 4(2): 1-7.

11. Poggio F, Del Mastro L, Bruzzone M, Ceppi M, Razeti MG, Fregatti P, et al. Safety of systemic hormone replacement therapy in breast cancer survivors: a systematic review and meta-analysis. Breast Cancer Res Treat. (2022) 191(2):269-275.

12. Bhardwaj P, Au CC, Benito-Martin A, Ladumor H, Oshchepkova S, Moges R, Brown KA. Estrogens and breast cancer: Mechanisms involved in obesity-related development, growth and progression. The Journal of steroid biochemistry and molecular biology. (2019) 189, 161–170.

13. Sartorius CA, Hanna CT, Gril B, Cruz H, Serkova NJ, Huber KM,, et al. Estrogen promotes the brain metastatic colonization of triple negative breast cancer cells via an astrocyte-mediated paracrine mechanism. Oncogene. (2016) 35(22):2881-92.

14. Chakraborty B, Byemerwa J, Shepherd J, Haines CN, Baldi R, Gong W, et al. Inhibition of estrogen signaling in myeloid cells increases tumor immunity in melanoma. J Clin Invest. (2021) Dec 1;131(23):e151347.

15. San Francisco IF, Rojas PA, DeWolf WC, Morgentaler A. Low free testosterone levels predict disease reclassification in men with prostate cancer undergoing active surveillance. BJU Int. (2014) 114(2):229-35.

16. Tu H, Gu J, Meng QH, Kim J, Strom S, Davis JW, et al. Low serum testosterone is associated with tumor aggressiveness and poor prognosis in prostate cancer. Oncol Lett. (2017) 13(3):1949-1957.

17. Leitner BP, Siebel S, Akingbesote ND, Zhang X, Perry RJ.. Insulin and cancer: a tangled web. Biochem J. (2022) 479(5):583-607.

18. Bastos DB, Sarafim-Silva BAM, Sundefeld MLMM, Ribeiro AA, Brandão JDP, Biasoli ÉR, et al. Circulating catecholamines are associated with biobehavioral factors and anxiety symptoms in head and neck cancer patients. PLoS One. (2018) 13(8).

19. Elenkov IJ, Wilder RL, Chrousos GP, Vizi ES. The sympathetic nerve--an integrative interface between two supersystems: the brain and the immune system. Pharmacol Rev (2000) 52:595-638.

20. He XY, Ng D, Van Aelst L, Egeblad M. Stressing Out about Cancer Immunotherapy. Cancer Cell. (2019) 11;36(5):468-470.

21. Sherbet GV. Hormonal influences on cancer progression and prognosis. Vitam Horm (2005) 71:147-200.

22. Conforti F, Pala L, Bagnardi V, De Pas T, Martinetti M, Viale G, et al. Cancer immunotherapy efficacy and patients’ sex: a systematic review and meta-analysis. Lancet Oncol. (2108) 4, 1–10.

23. Ye Y, Jing Y, Li L, Mills GB, Diao L, Liu H, Han L. Sex-associated molecular differences for cancer immunotherapy. Nat Commun (2020) 11, 1779.

24. Su SC, Hsieh MJ, Yang WE, Chung WH, Reiter RJ, Yang SF. Cancer metastasis: mechanisms of inhibition by melatonin. J Pineal Res (2017) 62(1).

25. Zaki NF, Sabri YM, Farouk O, Abdelfatah A, Spence DW, Bahammam AS, Pandi-Perumal SR. Depressive symptoms, sleep profiles and serum melatonin levels in a sample of breast cancer patients. Nat Sci Sleep (2020) 12:135-49.

26. Pan Z, Choi S, Ouadid-Ahidouch H, Yang JM, Beattie JH, Korichneva I. Zinc transporters and dysregulated channels in cancers. Front Biosci (Landmark Ed). (2017) 22:623-643.

27. Hu MJ, He JL, Tong XR, Yang WJ, Zhao HH, Li GA, Huang F. Associations between essential microelements exposure and the aggressive clinicopathologic characteristics of papillary thyroid cancer. Biometals. (2021) 34(4):909-921.

28. Cai X, Wang C, Yu W, Fan W, Wang S, Shen N, et al. Selenium Exposure and Cancer Risk: an Updated Meta-analysis and Meta-regression. Sci Rep. (2016) 6:19213

29. Vinceti M, Filippini T, Del Giovane C, Dennert G, Zwahlen M, Brinkman M, et al. Selenium for preventing cancer. Cochrane Database Syst Rev. (2018) 1(1):CD005195.

30. Anderson JJ. Potential health concerns of dietary phosphorus: cancer, obesity, and hypertension. Ann N Y Acad Sci. (2013) 1301:1-8.

31. Yoshida T, Taguchi D, Fukuda K, Shimazu K, Inoue M, Murata K, Shibata H. Incidence of hypophosphatemia in advanced cancer patients: a recent report from a single institution. Int J Clin Oncol. (2017) 22(2):244-249.

32. Lee DH, Anderson KE, Harnack LJ, Folsom AR, Jacobs DR Jr. Heme iron, zinc, alcohol consumption, and colon cancer: Iowa Women's Health Study. J Natl Cancer Inst. (2004) 96(5):403-7.

33. Torti, S., Torti, F. Iron and cancer: more ore to be mined. Nat Rev Cancer (2013) 13, 342–355.

34. Aksan A, Farrag K, Aksan S, Schroeder O, Stein J. Flipside of the Coin: Iron Deficiency and Colorectal Cancer. Front Immunol. (2021) 12:635899.

35. Luo H, Zhang NQ, Huang J, Zhang X, Feng XL, Pan ZZ,, et al. Different forms and sources of iron in relation to colorectal cancer risk: a case-control study in China. Br J Nutr. (2019) 121(7):735-747.

36. Castiglioni S, Maier JA. Magnesium and cancer: a dangerous liason. Magnes Res. (2011) 24(3):S92-100.

37. Gorczyca AM, He K, Xun P, Margolis KL, Wallace JP, Lane D,, et al. Association between magnesium intake and risk of colorectal cancer among postmenopausal women. Cancer Causes Control. (2015) 26:1761-1769.

38. Lin J, Cook NR, Lee IM, Manson JE, Buring JE, Zhang SM. Total magnesium intake and colorectal cancer incidence in women. Cancer Epidemiol Biomarkers Prev. (2006) 15:2006-2009.

39. Dibaba D, Xun P, Yokota K, White E, He K. Magnesium intake and incidence of pancreatic cancer: the VITamins and Lifestyle study. Br J Cancer. (2015) 113:1615-1621.

40. Zheng W, Anderson KE, Kushi LH, Sellers TA, Greenstein J, Hong CP,, et al. A prospective cohort study of intake of calcium, vitamin D, and other micronutrients in relation to incidence of rectal cancer among postmenopausal women. Cancer Epidemiology, Biomarkers and Prevention (1998) 7(3):221–225.

41. Baron JA, Beach M, Mandel JS, van Stolk RU, Haile RW, Sandler RS, et al. Calcium supplements and colorectal adenomas. Polyp Prevention Study Group. Annals of the New York Academy of Sciences (1999) 889:138–145.

42. Baron JA, Beach M, Mandel JS, van Stolk RU, Haile RW, Sandler RS, et al. Calcium supplements for the prevention of colorectal adenomas. Calcium Polyp Prevention Study Group. New England Journal of Medicine (1999) 340(2):101–107.

43. Bonithon-Kopp C, Kronborg O, Giacosa A, Räth U, Faivre J. Calcium and fibre supplementation in prevention of colorectal adenoma recurrence: A randomized intervention trial. Lancet (2000) 356 (9238):1300–1306.

44. Terry P, Baron JA, Bergkvist L, Holmberg L, Wolk A . Dietary calcium and vitamin D intake and risk of colorectal cancer: A prospective cohort study in women. Nutrition and Cancer (2002) 43(1):39–46.

45. Lappe JM, Travers-Gustafson D, Davies KM, Recker RR, Heaney RP. Vitamin D and calcium supplementation reduces cancer risk: Results of a randomized trial. American Journal of Clinical Nutrition (2007) 85(6):1586–1591.

46. Park Y, Leitzmann MF, Subar AF, Hollenbeck A, Schatzkin A. Dairy food, calcium, and risk of cancer in the NIH-AARP Diet and Health Study. Archives of Internal Medicine (2009) 169(4):391–401.

47. Ahn J, Albanes D, Peters U, Schatzkin A, Lim U, Freedman M, et al. Dairy products, calcium intake, and risk of prostate cancer in the Prostate, Lung, Colorectal, and Ovarian Cancer Screening Trial. Cancer Epidemiology, Biomarkers and Prevention (2007) 16(12):2623–2630.

48. Machida-Montani A, Sasazuki S, Inoue M, Natsukawa S, Shaura K, Koizumi Y, et al. Association of Helicobacter pylori infection and environmental factors in non-cardia gastric cancer in Japan. Gastric Cancer. (2004) 7(1):46-53.

49. D'Elia L, Rossi G, Ippolito R, Cappuccio FP, Strazzullo P. Habitual salt intake and risk of gastric cancer: a meta-analysis of prospective studies. Clin Nutr. (2012) 31(4):489-98.

50. Lv M, Chen M, Zhang R, Zhang W, Wang C, Zhang Y, et al. Manganese is critical for antitumor immune responses via cGAS-STING and improves the efficacy of clinical immunotherapy. Cell Res (2020) 30, 966–979.

51. Hernroth B, Holm I, Gondikas A, Tassidis H. Manganese Inhibits Viability of Prostate Cancer Cells. Anticancer Res. (2018) 38(1):137-145.

52. Ramchandani D, Berisa M, Tavarez DA, Li Z, Miele M, Bai Y, et al. Copper depletion modulates mitochondrial oxidative phosphorylation to impair triple negative breast cancer metastasis. Nat Commun (2021) 12, 7311.

53. Liu YL, Bager CL, Willumsen, Ramchadani D, Kornhauser N, Ling L, et al. Tetrathiomolybdate (TM)-associated copper depletion influences collagen remodeling and immune response in the pre-metastatic niche of breast cancer. *npj* Breast Cancer (2021) 7, 108.

54. Micali S, Bulotta S, Puppin C, Territo A, Navarra M, Bianchi G, et al. Sodium iodide symporter (NIS) in extrathyroidal malignancies: focus on breast and urological cancer. BMC Cancer. (2014) 14:303.

55. Verheesen RH, Schweitzer CM Iodine deficiency, more than cretinism and goiter. Med Hypotheses (2008) 71, 645-8.

56. Rappaport J. Changes in Dietary Iodine Explains Increasing Incidence of Breast Cancer with Distant Involvement in Young Women. J Cancer. (2017) 8(2):174-177.

57. Manjer J, Sandsveden M, Borgquist S. Serum Iodine and Breast Cancer Risk: A Prospective Nested Case-Control Study Stratified for Selenium Levels. Cancer Epidemiol Biomarkers Prev (2020) 29(7):1335-1340.
